# 

**Published:** 1895-03

**Authors:** 


### CONTENTS.

ORIGINAL COMMUNICATIONS—
- Spastic Paralysis; Talipes Equino-Varus. By Mr. Edmon Owen, London, Eng.. 1
- Medical Examinations for Life Insurance Companies. By Jas. N. West, M.D., Toccoa, Ga. 7
- Nasal Obstruction and Catarrh. By Austin S. Tinsley, M.D., Augusta, Ga. 10
- The Antitoxin Treatment of Diphtheria. By L. F. Barker, M.D., Baltimore, Md. 13
- The Coal-Tar Derivatives. By J. W. Duncan, M.D., Atlanta, Ga. 17
- Placenta Previa. By P. Guntermann, M.D., Louisville, Ky. 20

SOCIETY REPORTS—

- Atlanta Medical Society 24

- Richmond Academy of Medicine and Surgery 27

CORRESPONDENCE—
- Treatment of Hemorrhoids by Iodoform in Ether 30

EDITORIAL—
- Volume Twelve 32
- A National Board of Health 32
- Ministerial Indorsements of Humbugs 34

SELECTIONS AND ABSTRACTS—

- Eye, Ear, Nose, and Throat 35

- General Medicine and Therapeutics 39

- Skin Diseases and Syphilis 44

- Obstetrics and Gynecology 50

- Surgery 56

BOOK REVIEWS 59

MEDICAL ITEMS 63

ADVERTISERS' INDEX TO ADVERTISEMENTS xii

CLASSIFIED ADVERTISING INDEX xxxi

MEDICAL ITEMS—Continued ix, x, xi

